# Ambulatory blood pressure parameters and their association with albuminuria in adolescents with type 1 diabetes mellitus

**DOI:** 10.1007/s00467-024-06416-3

**Published:** 2024-06-12

**Authors:** Jolanta Sołtysiak, Bogda Skowrońska, Katarzyna Maćkowiak-Lewandowicz, Andrzej Blumczyński, Kaczmarek Elżbieta, Danuta Ostalska-Nowicka, Jacek Zachwieja

**Affiliations:** 1 Department of Pediatric Nephrology and Hypertension, Poznan University of Medical Sciences, 27/33 Szpitalna St., 60-572 Poznan, Poland; 2 Pediatric Diabetes and Obesity, Poznan University of Medical Sciences, 60-572 Poznan, Poland; 3 Department of Bioinformatics and Computational Biology, Poznan University of Medical Sciences, 60-572 Poznan, Poland

**Keywords:** Diabetes mellitus, Ambulatory blood pressure monitoring, Hypertension, Diabetic kidney disease, Albuminuria, Pediatrics, Vascular disease

## Abstract

**Background:**

This study aimed to evaluate the blood pressure (BP) status, including arterial stiffness parameters, hemodynamic indicators, circadian profile, and its association with albuminuria in adolescents with type 1 diabetes mellitus (DM1).

**Methods:**

The analysis included 46 patients, with diabetes duration of 7.38 ± 3.48 years. Ambulatory blood pressure monitoring (ABPM) was conducted using an oscillometric device, the Mobil-O-Graph, which is a Pulse Wave Analysis Monitor.

**Results:**

Hypertension (HT) was diagnosed in 31 adolescents (67% of patients), primarily due to isolated nocturnal BP (21 cases, 68% of HT cases). The HT group exhibited significantly increased diastolic load (DL). Pulse wave velocity (PWV, a measure of arterial stiffness) values showed a strong correlation with both peripheral systolic BP (*r* = 0.954) and central systolic BP (*r* = 0.838). Additionally, non-dipping status was found in 61% of the HT group. Urinary albumin excretion (UAE) was positively correlated with diastolic BP (particularly nocturnal) peripheral and central BP, DL, heart rate, augmentation index (AIx@75), and nocturnal total vascular resistance (TVR). Diastolic non-dippers exhibited a significant increase in UAE.

**Conclusions:**

Hypertension is a common complication in adolescents with type 1 diabetes mellitus, primarily caused by elevated nocturnal diastolic BP. Albuminuria is mainly associated with diastolic BP, especially during the nocturnal period and in cases of diastolic non-dipping status. The association of UAE with AIx@75 and nocturnal TVR suggests the presence of early-stage vascular disease in diabetic adolescents.

**Graphical abstract:**

**Figure:**
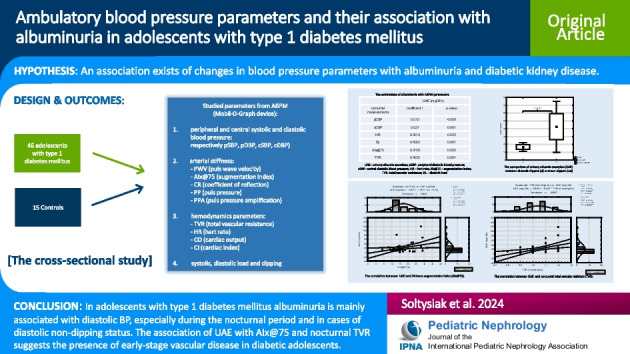
A higher resolution version of the Graphical abstract is available as Supplementary information

**Supplementary Information:**

The online version contains supplementary material available at 10.1007/s00467-024-06416-3.

## Introduction

Diabetes mellitus is a prevalent disorder affecting a substantial number of individuals worldwide, and diabetic kidney disease (DKD) is a leading cause of chronic kidney disease (CKD) [1]. DKD is a multifactorial condition influenced by various risk factors such as glycemic control, duration of diabetes, genetics, sex, cholesterol levels, and smoking [2]. Hypertension, a common comorbidity in individuals with diabetes, is a significant risk factor for the development and progression of microvascular complications, including cardiovascular and renal diseases [3]. Among patients with type 1 diabetes, the incidence of hypertension increases with disease progression, reaching 5% at 10 years, 33% at 20 years, and 70% at 40 years [4]. 

In addition to kidney disease development, diabetes contributes to hypertension through two other proposed mechanisms: extracellular fluid volume expansion and increased arterial stiffness [5]. Insulin and the elevated glucose load in hyperglycemia lead to extracellular fluid volume expansion and sodium retention, thereby raising blood pressure (BP) [5, 6]. Increased vascular stiffness, observed in diabetic patients, is attributed to protein glycation and atheromatous disease, accompanied by decreased activation of endothelial nitric oxide synthase and reduced nitric oxide (NO) levels, further promoting arterial stiffness [5]. The inappropriate activation of the renin–angiotensin–aldosterone system and mineralocorticoid receptor through insulin contributes to the reduced activation of endothelial nitric oxide synthase in diabetes [5]. 

Diabetes is associated with the loss of normal nocturnal blood pressure dipping, known as a “non-dipping BP profile,” defined as a nocturnal blood pressure decrease of less than 10% [3, 7]. Non-dipping BP is linked to a higher risk of nephropathy and adverse cardiovascular outcomes [3, 7]. Ambulatory blood pressure monitoring (ABPM), a 24-h method, is considered effective for assessing and managing hypertension, as well as evaluating the circadian blood pressure rhythm. ABPM has the potential to improve clinical outcomes for patients with cardiovascular risk factors [8]. Various devices are available for ABPM, some of which can non-invasively measure arterial stiffness and hemodynamic indicators. In a study involving 36 children with type 1 diabetes, the Mobil-O-Graph device demonstrated increased arterial stiffness parameters (augmentation index, reflection coefficient, and augmentation pressure) and decreased hemodynamic indicators (systolic volume and cardiac output) [9]. This indicates the potential impact of diabetes on vascular function and cardiac performance, making ABPM a valuable tool for assessing and managing these complications in diabetic patients.

Albuminuria is a valuable clinical indicator for early prediction of future cardiorenal damage [10]. It is closely related to the prevalence of hypertension and increasing albuminuria in diabetes. Blood pressure typically begins to rise within the normal range or shortly after moderately increased albuminuria appears [11]. Additionally, increased albuminuria is associated with the absence of nocturnal blood pressure decrease in non-dipping patients. In adolescents and young adults with type 1 diabetes mellitus (DM1), an increase in systolic blood pressure during sleep has been shown to precede the development of microalbuminuria. Conversely, when blood pressure decreases normally during sleep, the progression from normal albumin excretion to microalbuminuria appears to be less likely [12].

In adolescents with DM1, research on the relationship between albuminuria and hypertension, as well as arterial stiffness and hemodynamic changes, is limited [13, 14]. In contrast, studies involving adolescents with type 2 diabetes or adults often focus on the metabolic syndrome as the primary cause of hyperglycemia and increased blood pressure. Since adolescents with DM1 have relatively short diabetes durations, changes detected in this group may reflect early stages of cardiovascular injury. The early detection of vascular disturbances and appropriate blood pressure control are essential in preventing cardiorenal damage in this population. By focusing on these early indicators, healthcare providers can intervene promptly to minimize the risk of long-term complications.

The objective of the present study was to evaluate the blood pressure status, including the frequency of hypertension and the circadian profile assessed by ABPM in adolescents with type 1 diabetes mellitus. Next, we aimed to assess arterial stiffness parameters and hemodynamic indicators obtained from ABPM using the Mobil-O-Graph device and further, we analyzed the association between the studied parameters and albuminuria as an early predictor of cardiorenal damage.

## Methods

The analysis included a group of 46 adolescents with type 1 diabetes mellitus, with an average diabetes duration of 7.38 ± 3.48 years. These patients were admitted to the Department of Pediatric Diabetology, Auxology, and Obesity at Poznan University of Medical Sciences in Poland for diabetes assessment, including evaluation of hypertension. ABPM was performed on 77 adolescents suspected of having hypertension based on casual blood pressure measurements or in patients approaching adulthood.

**Fig. 1:**
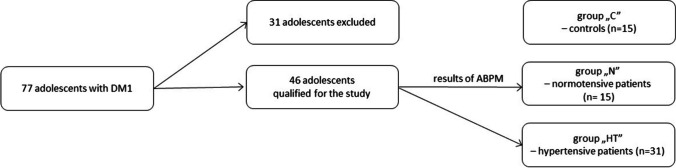
The flow diagram of the study methodology

Patients with a history of conditions affecting blood pressure, particularly heart and kidney diseases, or other serious diseases or factors influencing blood pressure, such as drug abuse or smoking, were excluded. Additionally, patients receiving antihypertensive therapy, those who were obese or overweight, and those with fewer than 40 valid recordings in ABPM were excluded (see Fig. 1).

All subjects maintained their normal sodium intake, and no medication other than insulin was taken. There were 12 patients with hypertension based on office blood pressure measurements. The mean office blood pressure among diabetic patients was 127/79 mmHg, while the mean height of the group was 173.92 ± 10.19 cm.

Ambulatory blood pressure monitoring (ABPM) using the Mobil-O-Graph device—the Pulse Wave Analysis Monitor (Mobil-O-Graph; IEM, Stolberg, Germany)—was initiated upon discharge from the hospital. The Mobil-O-Graph device incorporates the ARCSolver method (Austrian Institute of Technology, Vienna, Austria), which utilizes a transfer function to reconstruct the central or aortic pulse wave from brachial oscillometric pressure. Central data were recorded for approximately 10 s at the diastolic blood pressure level (± 5 mmHg) using a high-fidelity pressure sensor (MPX5050; Freescale, Tempe, Arizona) [15, 16]. Initially, the arm circumference of each participant was measured to determine the appropriate cuff size for ABPM. The ABPM measurements were programmed to occur every 20 min during the day (7 am to 11 pm) and every 30 min during the night (11 pm to 7 am). Parents and children were instructed to maintain a diary of daily activities during the ABPM measurement period. The cuff was placed on the non-dominant arm, and participants were advised to avoid vigorous physical exercise while wearing the ABPM device but to continue with their usual daily activities.

The classification of ambulatory blood pressure in adolescents was based on the guidelines published by Flynn et al. in 2022 [17]. Adolescents aged 13 years and older were classified as hypertensive (HT group) if their blood pressure was equal to or exceeded 125/75 mmHg during the 24-h period, 130/80 mmHg during the daytime period, or 110/65 mmHg during the nighttime period. For patients younger than 13 years of age (five cases), hypertension was recognized if the blood pressure was equal to or above the 95th percentile during the 24-h, daytime, or nighttime period [18]. Normal blood pressure was defined as being below the 90th percentile during the 24-h period, as well as during both daytime and nighttime periods.

The ABPM parameters and their clinical significance, obtained from Mobil-O-Graph, are presented in the supplementary material. Peripheral and central blood pressure, mean arterial pressure (MAP), and arterial stiffness indexes such as pulse wave velocity (PWV), augmentation index (AIx@75), reflection coefficient (CR), peripheral pulse pressure (pPP), central pulse pressure (cPP), and pulse pressure amplification (PPA) were evaluated. PWV was determined using a mathematical model that incorporated various parameters in pulse wave and wave separation analysis [19]. AIx@75 was calculated by determining the pressure difference between the reflection wave peak (P2) and the incident wave peak (P1), expressed as a percentage of pulse pressure [AIx@75 = (P2—P1)/PPc × 100] [19]. Hemodynamic parameters, including total vascular resistance (TVR), heart rate (HR), cardiac output (CO), and cardiac index (CI), were also studied. The systolic and diastolic blood pressure load, as well as the number of dippers and non-dippers, were assessed. Non-dippers were identified as individuals with a reduction in blood pressure of less than 10% from day to night in either systolic or diastolic blood pressure. Comparisons were made between systolic dippers and non-dippers, as well as diastolic dippers and non-dippers. Among the six diastolic non-dipping patients, four adolescents also had systolic non-dipping status.

Both office BP and ABPM parameters, as well as PWV values, are dependent on sex, age, and height. Therefore, standard deviation scores (SDS) for office systolic and diastolic BP, ABPM (peripheral systolic blood pressure [pSBP], peripheral diastolic blood pressure [pDBP], mean arterial pressure [MAP], heart rate [HR]), and PWV were calculated based on published centile charts [18, 20–22].

All study subjects underwent blood and urine tests. Urinary albumin excretion (UAE) was assessed through 24-h urine collection. Kidney function was estimated using the glomerular filtration rate (eGFR) based on the Filler formula, which incorporates cystatin C [23, 24]. Long-term glycemic control was evaluated using hemoglobin A1c (HbA1c) levels [25, 26]. Urinary sodium excretion (uNa), fractional sodium excretion (FNa), serum uric acid (UA), and neutrophil–lymphocyte ratio (NLR) as an inflammatory marker were also analyzed.

The control group consisted of 15 healthy children (with an equal number of males and females) recruited from adolescents hospitalized in the Department of Pediatric Nephrology and Hypertension, where hypertension was excluded based on ABPM.

The study was approved by the Bioethics Committee of Poznan University of Medical Sciences, Poland.

### Statistical analysis

The data are reported as the mean ± standard deviation. Continuous variables were tested using one-way analysis of variance (ANOVA) with the Scheffé post hoc test for comparisons among more than two groups. For comparisons involving two groups, the non-parametric Mann–Whitney test was applied. For comparisons among three or more groups, the non-parametric Kruskal–Wallis test was used. The association between two variables was assessed using Spearman’s rank correlation coefficient. The level of significance was set at *α* = 0.050.

The power of the test for assessing minimal significant differences between groups was also performed. In a pilot group of size 15 (the control group), we assumed that minimal significant differences should be at least 10 mg/24 h for urinary albumin excretion (UAE), 10 mmHg for systolic blood pressure, and 10 mmHg for diastolic blood pressure. The computed power of the tests for minimal significant differences between the compared groups (at least 10) was 0.75 (*α* = 0.050) with the minimal sample size of 15.

The association between sex and dippers/non-dippers in the studied subgroups was tested using Fisher’s exact test. Office casual systolic blood pressure (SBP) and diastolic blood pressure (DBP), peripheral SBP (pSBP), peripheral DBP (pDBP), and pulse wave velocity (PWV) were adjusted for sex, age, and height, and standard deviation scores (SDS) were calculated based on the corresponding growth charts.

Statistical analysis was performed using Statistica version 8 (StatSoft, Tulsa) and JAMOVI.

## Results

The analysis of blood pressure parameters obtained from ABPM (Table 1) showed that 67% of the patients (31 individuals) were diagnosed with arterial hypertension (HT), while 33% (15 patients) had normal blood pressure (*N*). In ten cases, office hypertension was confirmed by ABPM (ambulatory hypertension); two cases involved white coat hypertension (normal ABPM), while 21 cases of HT were newly diagnosed, including isolated nighttime hypertension (68% of hypertensive patients) and classified as masked hypertension.

**Table 1:** The demographic, clinical data, and ABPM results of the study groups and controls

	Controls (*n* = 15)	Normal BP group (*n* = 15; 33%)	Hypertensive group (*n* = 31; 67%)	*p*_HT/N_	*p*_HT/C_
Age (ys.)	15.32 ± 1.74	15.10 ± 2.62	16.60 ± 1.42	0.053	0.142
Age at onset (ys.)	na	8.63 ± 3.44	8.77 ± 3.80	0.913	na
Female/male	9/6	8/7	6/25	0.038	0.009
Height (cm)	170.50 ± 8.40	169.57 ± 8.24	176.03 ± 10.49	0.112	0.193
SDS of height	0.54 ± 0.84	0.84 ± 1.0	0.33 ± 1.30	0.190	0.571
BMI (kg/m^2^)	22.41 ± 3.17	22.96 ± 3.55	22.30 ± 3.36	0.823	0.992
Diabetes duration (ys.)	na	6.46 ± 3.10	7.83 ± 3.61	0.222	na
Office SBP (mmHg)	119.00 ± 6.31	124.46 ± 6.12	127.77 ± 9.60	0.271	0.022
SDS of office SBP	0.46 ± 0.69	1.06 ± 0.62	0.91 ± 0.77	0.549	0.115
Office DBP (mmHg)	73.82 ± 5.86	77.38 ± 4.31	79.32 ± 7.89	0.421	0.094
SDS of office DBP	1.19 ± 0.74	1.706 ± 0.519	1.69 ± 0.98	0.951	0.149
ABPM results					
pSBP (mmHg)	113.13 ± 6.07	112.87 ± 5.34	122.97 ± 5.00	< 0.001	< 0.001
SDS of pSBP	− 0.51 ± 0 .66	− 0.51 ± 0.87	0.5 5 ± 0.78	< 0.001	< 0.001
pDBP (mmHg)	65.80 ± 4.29	66.20 ± 4.71	72.61 ± 4.83	< 0.001	< 0.001
SDS of pDBP	− 0.37 ± 0.77	− 0.32 ± 0.91	0.84 ± 0.91	< 0.001	< 0.001
MAP (mmHg)	87.67 ± 4.69	87.60 ± 4.34	95.58 ± 3.62	< 0.001	< 0.001
SDS of MAP	0.75 ± 0.69	0.75 ± 0.79	1.90 ± 0.80	< 0.001	< 0.001
SL (%)	11.33 ± 6.16	13.53 ± 10.68	19.81 ± 15.06	0.283	0.101
DL (%)	11.73 ± 10.57	14.27 ± 10.05	27.94 ± 15.92	0.008	0.001
Number of patients with isolated nighttime HT	na	na	21 (68%)	na	na
Number of patients with isolated daytime HT	na	na	3 (10%)	na	na
Number of patients with night- and daytime HT	na	na	7 (22%)	na	na
cSBP (mmHg)	113.53 ± 6.92	113.29 ± 6.39	122.34 ± 6.74	< 0.001	< 0.001
cDBP	68.47 ± 4.67	66.63 ± 3.81	75.03 ± 4.58	< 0.001	< 0.001
Laboratory results					
UAE (mg/24 h)	11.43 ± 3.81	12.65 ± 8.92	24.13 ± 26.60 median (Q1–Q3) median = 12.30 lower quartile = 7.50 upper quartile = 20.45	0.264	0.422
eGFR (ml/min/1.73 m^2^)	100.49 ± 12.21	115.98 ± 19.74	112.47 ± 28.72	0.909	0.623
HbA1c (%)	5.06 ± 0.19	7.73 ± 1.35	8.33 ± 2.09	0.578	0.002
UA (mg/dl)	5.44 ± 1.34	4.57 ± 1.16	5.24 ± 1.32	0.265	0.93
Chol (mg/dl)	145.17 ± 22.93	195.87 ± 30.81	187.27 ± 50.83	0.822	0.112
TG (mg/dl)	78.17 ± 32.18	88.93 ± 39.30	106.21 ± 75.39	0.691	0.613
mNa (mmol/24 h)	127.23 ± 47.56	166.27 ± 71.72	154.81 ± 76.88	0.72	0.089
FNa	0.56 ± 0.19	0.70 ± 0.25	0.67 ± 0.38	0.963	0.791
NLR	2.33 ± 0.94	2.22 ± 1.04	1.91 ± 1.03	0.578	0.236
INS (j/kg)	na	18.74 ± 5.17	21.71 ± 6.12	0.25	na

Data were presented as *M* ± *SD* or absolute frequencies of occurrence. For continuous data Student t or Mann–Whitney *U* tests were performed, whereas for categorical data *χ*^2^ tests of independence were used. *BP* blood pressure, *HT* hypertension, *N* normal BP group, *C* controls, *ABPM* ambulatory blood pressure monitoring, *SBP* systolic blood pressure, *DBP* diastolic blood pressure, *pSBP* peripheral systolic blood pressure, *pDBP* peripheral diastolic blood pressure, *MAP* mean arterial pressure, *cSBP* central systolic blood pressure, *cDBP* central diastolic blood pressure, *SL* systolic load, *DL* diastolic load, *NLR* neutrophil–lymphocyte ratio, *SDS* standard deviation scores, *mNa* urinary sodium, *INS* basal insulin, *na* not applicable

**Fig. 2:**
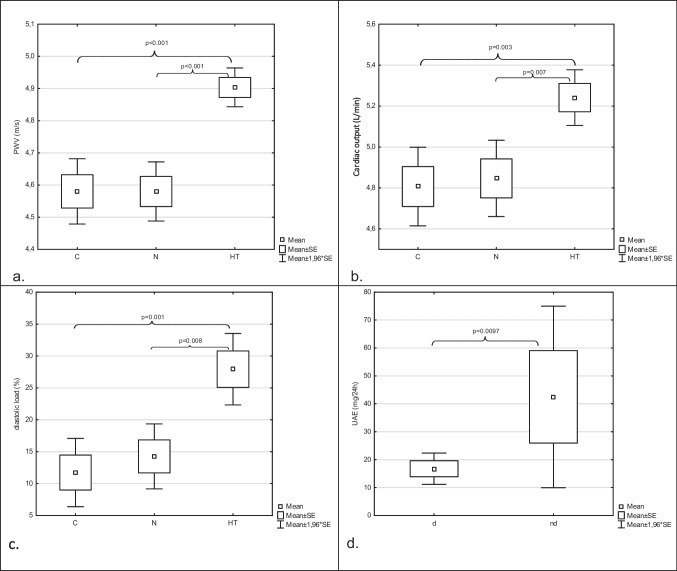
**a** The comparison of pulse wave velocity (PWV) between subgroups according to blood pressure; **b **the comparison of cardiac output (CO) between subgroups according to blood pressure; **c** the comparison of diastolic load (DL) between subgroups according to blood pressure; **d** the comparison of urinary albumin excretion (UAE) between diastolic non-dippers (nd) and dippers (d); C, control group; N, normotensive patients; HT, hypertensive patients

In the HT group, there was a significant increase in pulse wave velocity (PWV), cardiac output (CO), and diastolic load (DL) compared to patients with normal blood pressure and the control group (Table 2, Fig. 2). It is worth noting that after computing the standard deviation scores for PWV, the differences between the HT group and both the control and normal groups lost statistical significance. However, the direction of the association remained unchanged, with the HT group showing higher standard deviation scores compared to the control and normal groups. A similar outcome occurred after standardizing CO to cardiac index (CI). The direction of the relationship remained the same, with the HT group showing higher CI values, but the association became statistically non-significant. Other parameters related to arterial stiffness, hemodynamic indicators, and laboratory results did not show significant differences among the study groups. The PWV values showed a positive correlation with peripheral systolic BP (pSBP; *r* = 0.954; *p* < 0.001), peripheral diastolic BP (pDBP; *r* = 0.418; *p* = 0.006), central systolic BP (cSBP; *r* = 0.838; *p* < 0.001), central diastolic BP (cDBP; *r* = 0.500; *p* = 0.001), mean arterial pressure (MAP; *r* = 0.800; *p* < 0.001), and CO (*r* = 0.675; *p* < 0.001).

**Table 2:** The arterial stifness, hemodynamic parameters in studied groups and controls

	Controls (*n* = 15)	Normal BP group (*n* = 15; 33%)	Hypertensive group (*n* = 31; 67%)	p_HT/N_	p_HT/C_
Arterial stiffness					
PWV (m/s)	4.58 ± 0.20	4.58 ± 0.18	4.90 ± 0.17	< 0.001	< 0.001
SDS of PWV	− 1.01 ± 0.66	− 0.93 ± 0.78	− 0.37 ± 1.16	0.055	0.104
AIx@75 (%)	18.60 ± 5.21	18.67 ± 8.26	15.90 ± 5.73	0.396	0.411
CR (%)	56.39 ± 5.78	58.81 ± 5.69	54.72 ± 5.59	0.085	0.661
PPp (mmHg)	47.47 ± 4.63	46.60 ± 5.12	50.39 ± 6.30	0.113	0.271
PPc (mmHg)	0.07 ± 5.05	44.40 ± 6.01	47.31 ± 8.58	0.454	0.623
PPA	1.06 ± 0.08	1.06 ± 0.09	1.07 ± 0.09	0.827	0.862
Hemodynamics					
TVR (s*mmHg/mL)	1.11 ± 0.08	1.11 ± 0.09	1.09 ± 0.06	0.853	0.694
HR (bpm/min)	76.53 ± 10.81	75.80 ± 8.77	78.13 ± 7.83	0.707	0.853
SDS of HR	− 0.15 ± 0.85	− 0.22 ± 0.61	0.25 ± 0.65	0.025	0.085
CO (L/min)	4.81 ± 0.38	4.85 ± 0.37	5.24 ± 0.37	0.007	0.003
CI (L/min/m^2^)	2.76 ± 0.30	2.77 ± 0.29	2.89 ± 0.27	0.463	0.384
Dipping status					
Systolic dipping (%)	11.86 ± 4.89	10.16 ± 4.60	9.10 ± 3.90	0.743	0.141
Diastolic dipping (%)	17.23 ± 6.37	16.93 ± 6.90	14.71 ± 7.77	0.614	0.53
Non-dippers/dippers*	4/11 (33%/67%)	5/10 (33%/67%)	19/12 (61%/39%)	0.116	0.057
Diastolic non-dippers	2 (13%)	1 (7%)	5 (16%)	0.657	> 0.999
Systolic non-dippers	4 (27%)	4 (33%)	18 (58%)	0.546	0.260

Data were presented as *M* ± *SD* or absolute frequencies of occurrence. For continuous data Student *t* or Mann–Whitney *U* tests were performed, whereas for categorical data *χ*^2^ tests of independence or Fisher’s independence test were used. *Normal BP* normal blood pressure, *cSBP* central systolic blood pressure, *cDBP* central diastolic blood pressure, *PWV* pulse wave velocity, *AIx@75* augmentation index, *CR* coefficient of reflection, *pPP* peripheral pulse pressure, *cPP* central pulse pressure, *PPA* pulse pressure amplification, *TVR* total vascular resistance, *HR* heart rate, *CO* cardiac output, *CI* cardiac index, *SDS* standard deviation scores; *number (percentage) of non-dippers/dippers due to lack of fall in systolic or diastolic blood pressure; *na* not applicable

Among diabetic patients, there were the highest number of systolic non-dippers (*n* = 22), but diastolic non-dipping status significantly increased albuminuria (Table 3). The analysis of dipping/non-dipping status did not reveal any significant differences in blood pressure and laboratory parameters between systolic non-dippers and dippers. However, a significant increase in urinary albumin excretion (UAE) was observed in diastolic non-dippers compared to dippers (Table 3, Fig. 2). No differences were observed in other analyzed parameters and laboratory results.

**Table 3:** The comparison of albuminuria between dippers and non-dippers

Parameters	Systolic dippers (*n* = 24)	Systolic non-dippers (*n* = 22)	*p*
UAE (mg/24 h)	13.15 ± 8.84	26.91 ± 29.23	0.061
	Diastolic dippers (*n* = 40)	Diastolic non-dippers (*n* = 6)	
UAE (mg/24 h)	16.76 ± 16.83	42.50 ± 40.62	0.0097

Systolic non-dippers—due to lack of fall in systolic blood pressure

Diastolic non-dippers—due to lack of fall in diastolic blood pressure

The association of albuminuria with the studied parameters and laboratory results showed that the HT group had the highest albuminuria excretion, although it was not statistically significant compared to the *N* group and controls (Table 1).

**Fig. 3:**
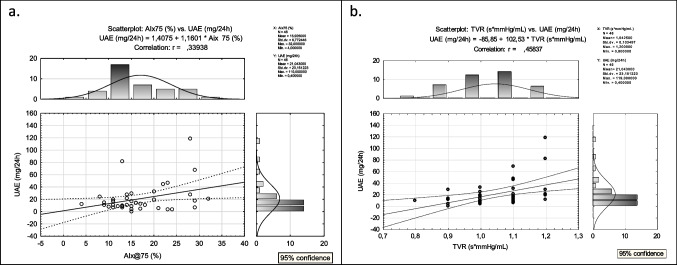
**a** The correlation between urinary albumin excretion (UAE) and a 24-h augmentation index (Aix@75); **b** the correlation between UAE and nocturnal total vascular resistant (TVR)

The study revealed a positive correlation between albuminuria and 24-h diastolic blood pressure (both peripheral and central), as well as heart rate (HR), augmentation index (AIx@75), and diastolic load (DL) (Table 4, Fig. 3). During the daytime, albuminuria showed a positive association with diastolic BP (both peripheral and central) and DL. However, during the nighttime, albuminuria additionally showed a positive association with HR, AIx@75, and total vascular resistance (TVR). Interestingly, the correlations between UAE and nocturnal diastolic BP parameters (pDBP, cDBP, and DL) showed stronger associations (*r* between 0.50 and 0.70) than daytime correlations. The calculation of correlation coefficients with standard deviation scores of peripheral DBP and HR did not cause any substantial changes except for 24-h HR, which became statistically insignificant after standardization.

**Table 4:** The correlations of albuminuria with parameters obtained from Mobil-O-Graph

	UAE (mg/24 h)	
24-h measurements	Pearson’s correlation coefficient *r*	*p*-value
pDBP	0.418	0.007
SDS pDBP	0.39	0.006
cDBP	0.402	0.010
HR	0.322	0.043
SDS HR	0.25	0.082
AIx@75	0.339	0.032
DL	0.447	0.003
Daytime measurements		
pDBP	0.324	0.041
SDS pDBP	0.31	0.032
cDBP	0.398	0.011
DL	*r* = 0.361	*p* = 0.026
Nocturnal measurements		
pDBP	0.572	< 0.001
SDS pDBP	0.50	< 0.001
cDBP	0.527	0.001
HR	0.3513	0.033
SDS HR	0.30	0.037
DL	0.5563	0.001
AIx@75	0.3759	0.022
TVR	0.4632	0.004

*UAE* urinary albumin excretion, *pDBP* peripheral diastolic blood pressure, *cDBP* central diastolic blood pressure, *HR* hart rate, *AIx@75* augmentation index, *TVR* total vascular resistance, *DL* diastolic load, *SDS* standard deviation scores

There were no significant correlations between albuminuria and PWV or other laboratory results. Glycated hemoglobin levels showed a positive correlation with nocturnal central DBP (*r* = 0.331). Moreover, two patients with hyperfiltration (eGFR of 190 and 183 ml/min/1.73 m^2^) both had hypertension. Other patients demonstrated an eGFR equal to or below 150 ml/min/1.73 m^2^. Moderate albuminuria (between 30 and 300 mg/24 h) was observed in six patients, five of whom had hypertension.

## Discussion

The findings of this study indicate that hypertension is a common complication among adolescents with type 1 diabetes mellitus (DM1), with a prevalence of 67% in the present study. In 21 cases (68%), hypertension was caused by isolated nighttime hypertension, while non-dippers were diagnosed in 19 cases (61% of hypertensive patients).

The prevalence of hypertension is higher than previously estimated. This may be due to the selected group, which included adolescents suspected of having hypertension, as well as the use of a new classification system according to Flynn et al., published in 2022 [17]. Earlier evaluations, which reported a hypertension prevalence of 19.4% in diabetic youth under 18 years of age, were based on casual blood pressure measurements and may have underestimated the prevalence of hypertension [4, 16]. Studies that employed ABPM in diabetic children showed a prevalence of hypertension reaching 33% [27]. In a study by Morić et al. of 201 children with T1D who had had diabetes for over 1 year, 15 children (7.5%) were found to be hypertensive using office blood pressure measurements, and only ten children (5%) were hypertensive according to ABPM. However, the 24-h systolic and diastolic blood pressure, as well as nighttime systolic and diastolic BP were significantly higher compared to reference values [28].

In the present study, casual hypertension was confirmed by ABPM in ten cases, while in two cases it was excluded by ABPM. In 21 cases, hypertension was newly diagnosed as masked hypertension, particularly due to isolated nighttime hypertension. In a study by Lee et al., nocturnal hypertension was found in 69% of hypertensive adolescents [29]. In the study by Gourgari et al., nocturnal hypertension was diagnosed in 36% of diabetic adolescents, while abnormal dipping occurred in 48% [14]. In the study by Morić et al., the percentage of non-dippers was 74.1% [28].

Furthermore, the present study demonstrated that among all the parameters obtained from ABPM using the Mobil-O-Graph device, the PWV, CO, and DL were significantly increased in hypertensive patients. The differences in absolute PWV values showed the highest level of statistical significance, with cardiac output also showing a significant increase. However, differences calculated with standardized variables (standard deviation scores of PWV and cardiac index) did not reach statistical significance. The theory suggests a positive association between both PWV and CO/CI with diabetes and hypertension [14, 30, 31]. Therefore, the lack of significance in standardized outcomes could likely be attributed to compromised statistical power due to the small sample size.

A previous study utilizing the Mobil-O-Graph device in 36 diabetic children (mean age 11.9 ± 3.2 years) did not find any increase in PWV, although other indicators of arterial stiffness were elevated. This study reported an increase in augmentation index (AIx@75), reflection coefficient, and augmentation pressure [9]. The cardiac output was even significantly lower in diabetic children. However, these patients did not show hypertension; their mean systolic BP was similar to that of the control group, while their mean diastolic BP was even lower. These patients were younger (mean age 11.9 ± 3.2 years), and there is no information available about the duration of diabetes.

In diabetic adults, it was demonstrated that PWV calculated from tonometry waveforms was significantly increased in different arteries, reflecting elevated central and peripheral artery stiffness [32]. In diabetic adolescents, Gourgari et al. found that sleep systolic BP, diastolic BP, and mean arterial pressure (MAP) were significantly associated with PWV [14]. In the present study, PWV also showed correlations with systolic and diastolic BP (both peripheral and central), as well as MAP and cardiac output. Interestingly, the strongest correlation was with peripheral systolic BP (*r* = 0.954).

Regarding increased CO, earlier studies demonstrated that in childhood, during an early phase of elevated BP, there was a hemodynamic profile of high cardiac output with normal peripheral resistance [33]. It was shown that in young adulthood and obese children, there is heightened adrenergic activity, which can increase CO [30, 34]. In diabetes, adrenergic activity was found to closely correlate with the degree of diabetes control [31]. Additionally, recovery from hypoglycemia, which often occurs in DM1, is also dependent on the adrenergic response; however, this aspect was not assessed in this study [35].

The analysis of other potential mechanisms contributing to hypertension in diabetes, such as sodium retention and diabetic kidney disease (DKD), did not reveal any associations with increased blood pressure. Daily urinary sodium excretion did not show any significant differences between the studied subgroups. Additionally, the insulin dose, which could potentially be related to sodium retention, did not correlate with sodium excretion. We also assessed the relationship between hypertension and the neutrophil-to-lymphocyte ratio (NLR), a hematological parameter for systemic inflammation and stress, which can also be influenced by diabetes. However, we did not find any significant relationship [36].

The analysis of the association between albuminuria and hypertension in hypertensive patients did not reveal a straightforward relationship. Although the hypertensive group exhibited the highest urinary albumin excretion, it was not statistically significant. No significant correlations were found between pulse wave velocity (PWV), cardiac output (CO), and albuminuria. However, further analysis revealed that increased albuminuria was strongly associated with both central and peripheral diastolic BP and diastolic load. Notably, nighttime measurements showed a stronger association with albuminuria than daytime results.

In a study by Gourgari et al. involving diabetic adolescents, sleep diastolic blood pressure, load, and mean arterial pressure (MAP) also positively correlated with albuminuria [14]. Morić et al. found correlations between albuminuria and daytime and nighttime diastolic BP [28]. They identified age and nighttime diastolic blood pressure variability as predictors of high-normal albuminuria, while nighttime diastolic blood pressure was a strong predictor of microalbuminuria. Additionally, patients with microalbuminuria had significantly higher 24-h, daytime, and nighttime diastolic blood pressure compared to normoalbuminuric subjects. These authors concluded that there is a clear connection between blood pressure, especially diastolic blood pressure, and incipient nephropathy, which was also demonstrated in the present study.

Furthermore, in this analysis, glycated hemoglobin levels showed a positive correlation with nocturnal central diastolic blood pressure, which may indicate an association between increased diastolic blood pressure and poor diabetes control. In our study, we found only six patients with moderate albuminuria. Five of them had hypertension, confirming that increased albuminuria and hypertension may occur concurrently and may reflect vascular disturbances in type 1 diabetes mellitus.

Moreover, in the present study, non-dipping status, particularly the absence of a nocturnal decrease in diastolic blood pressure, resulted in a significant increase in albuminuria. Patients who exhibited systolic non-dipping status showed a mild, but not statistically significant, increase in albuminuria. Non-dipping status was prevalent in hypertensive patients with diabetes, indicating its importance in diabetic adolescents.

Previous studies have also demonstrated a link between nocturnal blood pressure, particularly non-dipping status, and albuminuria in diabetic children [29, 37]. In adults, non-dipping status in DKD has been extensively studied as a risk factor for cardiovascular events [3, 7].

Interestingly, this study found that albuminuria showed a positive correlation with the augmentation index (AIx@75), nocturnal total vascular resistance (TVR), and heart rate (HR). High TVR is associated with mortality, heart failure, and cardiovascular events [38]. Another study utilizing the Mobil-O-Graph device reported a higher TVR in patients with peripheral arterial disease (PAD), which is atherosclerosis of the extremities and is associated with cardiovascular events [39]. While TVR did not show a significant increase in the hypertensive or non-dipping groups in the present study, the positive correlation between nocturnal TVR and albuminuria confirms its association with vascular disease.

AIx@75 provides an indirect measurement of arterial stiffness [40, 41]. Several studies have reported the clinical significance of AIx@75, which is associated with an increased risk of cardiovascular events and all-cause mortality [42]. AIx@75 can predict clinical cardiovascular events independent of peripheral pressures [42]. It is recognized as a useful marker of arterial stiffness in patients with PAD [43]. Although AIx@75 did not significantly increase in the hypertensive or non-dipping groups in this study, the positive correlations between AIx@75 and albuminuria further support its importance in vascular disturbances.

The limitation of our study is the low number of patients, particularly those with normal blood pressure and moderate albuminuria. This study was conducted among adolescents with relatively short diabetes duration and a low risk of chronic complications. As a result, the number of patients with moderate albuminuria or proteinuria was low, which could reflect early stages of cardiovascular injury.

Additionally, a significant statistical difference in the number of males and females between the hypertension and normal + control groups was found, which could potentially impact the results of further comparisons. However, we tried to minimize this by adjusting the most important variables according to appropriate growth charts.

The relationship between albuminuria and AIx@75, as well as total vascular resistance (TVR) in adolescents, needs further evaluation in larger samples. Additionally, the association between daily sodium intake and insulin dose with hypertension requires more detailed assessment. This cross-sectional study calls for further research to validate our results and investigate whether interventions targeting nighttime blood pressure could decrease the risk of cardiovascular disease.

## Conclusions

Hypertension is a common complication in adolescents with type 1 diabetes mellitus (DM1), primarily due to elevated nocturnal blood pressure. The presence of albuminuria is associated with an increase in diastolic blood pressure, particularly nocturnal diastolic blood pressure, diastolic non-dipping status, as well as augmentation index (AIx@75) and nocturnal total vascular resistance (TVR), which indicate early stages of vascular disease. Ambulatory blood pressure monitoring (ABPM) is necessary for the early detection of nocturnal blood pressure disturbances in diabetic adolescents. Proper control of nocturnal diastolic blood pressure and maintaining a normal circadian rhythm of blood pressure may be crucial in preventing cardiorenal damage.

### Supplementary Information

Below is the link to the electronic supplementary material.

Graphical abstract (PPTX 201 KB)
