# Supplementary material for: Ambulatory blood pressure parameters and their association with albuminuria in adolescents with type 1 diabetes mellitus
Source: Pediatr Nephrol. 2024 Jun 12;39(10):3037–47. doi: 10.1007/s00467-024-06416-3 (PMC11349841; doi:10.1007/s00467-024-06416-3)
Supplement: Supplementary file 1 — Graphical abstract (PPTX 201 KB) [file 467_2024_6416_MOESM1_ESM.pptx]

## Slide 1
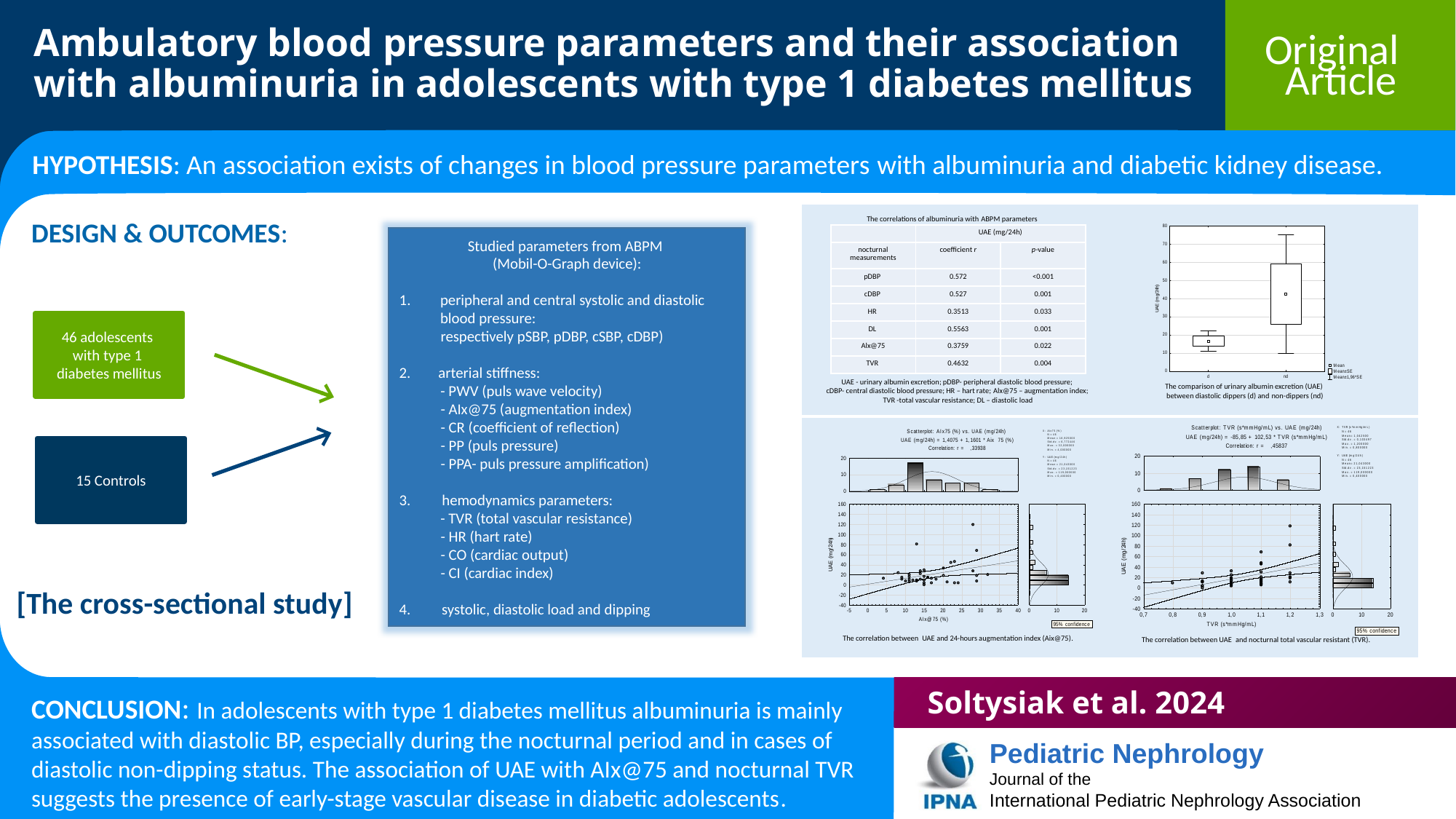

Ambulatory blood pressure parameters and their association with albuminuria in adolescents with type 1 diabetes mellitus
HYPOTHESIS: An association exists of changes in blood pressure parameters with albuminuria and diabetic kidney disease.
| |
| --- |
The correlations of albuminuria with ABPM parameters
DESIGN & OUTCOMES:
| | UAE (mg/24h) | |
| --- | --- | --- |
| nocturnal measurements | coefficient r | p-value |
| pDBP | 0.572 | <0.001 |
| cDBP | 0.527 | 0.001 |
| HR | 0.3513 | 0.033 |
| DL | 0.5563 | 0.001 |
| Alx@75 | 0.3759 | 0.022 |
| TVR | 0.4632 | 0.004 |
Studied parameters from ABPM
(Mobil-O-Graph device):
peripheral and central systolic and diastolic blood pressure:
 respectively pSBP, pDBP, cSBP, cDBP)
2. arterial stiffness:
 - PWV (puls wave velocity)
 - AIx@75 (augmentation index)
 - CR (coefficient of reflection)
 - PP (puls pressure)
 - PPA- puls pressure amplification)
3. hemodynamics parameters:
 - TVR (total vascular resistance)
 - HR (hart rate)
 - CO (cardiac output)
 - CI (cardiac index)
4. systolic, diastolic load and dipping
46 adolescents
with type 1
diabetes mellitus
UAE - urinary albumin excretion; pDBP- peripheral diastolic blood pressure;
cDBP- central diastolic blood pressure; HR – hart rate; Alx@75 – augmentation index;
TVR -total vascular resistance; DL – diastolic load
The comparison of urinary albumin excretion (UAE)
 between diastolic dippers (d) and non-dippers (nd)
| |
| --- |
15 Controls
[The cross-sectional study]
The correlation between UAE and 24-hours augmentation index (Aix@75).
The correlation between UAE and nocturnal total vascular resistant (TVR).
Soltysiak et al. 2024
CONCLUSION: In adolescents with type 1 diabetes mellitus albuminuria is mainly associated with diastolic BP, especially during the nocturnal period and in cases of diastolic non-dipping status. The association of UAE with AIx@75 and nocturnal TVR suggests the presence of early-stage vascular disease in diabetic adolescents.
